# Dectin-1 Polymorphism: A Genetic Disease Specifier in Autism Spectrum Disorders?

**DOI:** 10.1371/journal.pone.0137339

**Published:** 2015-09-09

**Authors:** Meriem Bennabi, Richard Delorme, José Oliveira, Catherine Fortier, Mohamed Lajnef, Wahid Boukouaci, Jean-Paul Feugeas, François Marzais, Alexandru Gaman, Dominique Charron, Bijan Ghaleh, Rajagopal Krishnamoorthy, Marion Leboyer, Ryad Tamouza

**Affiliations:** 1 INSERM, U1160, Hôpital Saint Louis, Paris, France; 2 INSERM, U955, Psychiatrie Génétique, Créteil, France; 3 Fondation FondaMental, Créteil, France; 4 DHU Protect, Service de Psychiatrie de l’Enfant et de l’Adolescent, Hôpital Robert Debré, Paris, France; 5 Département de Génétique Humaine et Fonctions Cognitives, Institut Pasteur, Paris, France; 6 Laboratoire Jean Dausset and LabEx Transplantex, Hôpital Saint Louis, Paris, France; 7 INSERM, U1137, Hôpital Bichat, Paris, France; 8 Université Paris Diderot, Sorbonne Paris-Cité, Paris, France; 9 Centre de Ressources Biologiques, Hôpitaux Universitaires Henri Mondor, Créteil, France; 10 AP-HP, Pôle de Psychiatrie, DHU PePSY, Hôpitaux Universitaires Henri Mondor, Créteil, France; 11 Université Paris-Est, Faculté de Médecine, Créteil, France; The George Washington University, UNITED STATES

## Abstract

**Introduction:**

In autism spectrum disorders (ASD), complex gene-environment interactions contribute to disease onset and progress. Given that gastro-intestinal dysfunctions are common in ASD, we postulated involvement of microbial dysbiosis in ASD and investigated, under a case-control design, the influence of DNA polymorphisms in the *CLEC7A* gene that encodes a pivotal fungal sensor, Dectin-1.

**Material and methods:**

DNAs from 478 ASD patients and 351 healthy controls (HC) were analyzed for the *CLEC7A rs*16910631G/A and *rs*2078178 A/G single nucleotide polymorphisms (SNPs). Differences in the distribution of allele, genotype and haplotype by Chi-square testing and nonparametric analysis by Kruskal-Wallis/Mann–Whitney tests, where appropriate, were performed. The free statistical package R.2.13 software was used for the statistical analysis.

**Results:**

We found that the *CLEC7A rs*2078178 G allele and GG genotype were more prevalent in HC as compared to ASD but failed to reach statistical significance for the latter (pc = 0.01, 0.06 respectively). However, after phenotype-based stratification, the *CLEC7A rs*2078178 G allele and GG genotype were found to be significantly more frequent in the Asperger group as compared to other ASD subsets (pc = 0.02, 0.01), a finding reinforced by haplotype analysis (*rs*2078178/*rs*16910631 G-G/G-G) (pc = 0.002). Further, intellectual quotient (IQ)-based stratification of ASD patients revealed that IQ values increase linearly along the *CLEC7A rs*2078178 AA, AG and GG genotypes (p = 0.05) and in a recessive manner (GG *vs*. AA+AG p = 0.02), further confirmed by haplotype distribution (*CLEC7A rs*2078178-16910631; A-G/A-G, A-G/G-G and G-G/G-G, p = 0.02, G-G/G-G *vs*. others, p = 0.01).

**Conclusion:**

Our data suggest that the genetic diversity of *CLEC7A* gene influences the ASD phenotype by behaving as a disease specifier and imply that the genetic control of innate immune response could determine the ASD phenotype.

## Introduction

Autism spectrum disorders (ASD) are a heterogeneous group of neurodevelopmental disorders characterized by impairments in social interactions and communication with a restricted repertoire of interests, behavior and activities [1]. Substantial diversity in symptoms and severity of these manifestations led to the diagnostic classification of ASD into three major subtypes namely, Asperger, classical form and pervasive developmental disorders not otherwise specified (PDD-NOS), the important difference between the subtypes being the intellectual quotient (IQ) values [2]. While the heritability of ASD was estimated to be between 40 to 70% in twin studies, in molecular studies of unrelated individuals only 0.5% to 20% of ASD subjects have alterations in genes involved in neuronal and synaptic homeostasis [3, 4]. Such “heritability gap” between twin studies and studies of unrelated patient populations highlighted the prominent influence of environment on genetic liability in the etiology of ASD [5]. In this regard, prior studies reporting on altered innate immune response in ASD are of particular interest in that the innate immune system is the front line defense against environmental infectious stressors. Evidences supporting the immune dysfunction in ASD are several but to cite a few: i) ASD association with single nucleotide polymorphisms (SNPs) located in the major histocompatibility complex (MHC), in particular with IQ variations in patients [6]; ii) Significant correlation between maternal viral/bacterial infections and diagnosis of ASD in the offsprings [7,8,9]; iii) Genetic association of macrophage migration inhibitory factor (MIF) gene polymorphism with behavioral components of ASD [10]; iv) Genetically determined raised circulating levels of HLA-G (with consequent inefficient anti infectious response) conferring raised risk for ASD [11]; v) Association between family history of autoimmune diseases and increased risk of ASD in children [12, 13, 14, 15]; vi) Link between gut microbiota dysbioses and ASD on the one hand [16, 17] and the host immune system on the other [18, 19, 20]; vii) Parallels between peripheral cytokine profiles and changes in behavioral symptoms following immune insults in a subset of ASD children with persistent gastrointestinal symptoms [21] and viii) relationship between the history of intense antibiotic administration (potential cause of gut dysbioses) during early childhood and raised incidence of ASD [22, 23].

Indeed gastrointestinal (GI)-tract dysfunctions are common in children with ASD (23–70%). These ASD children with GI disorders (ASD^GI^) have chronic inflammation with nodular lymphoid hyperplasia, enterocolitis and mucosal infiltration by GI-tract immune cells [24] and the severity of the GI symptoms often paralleled that of ASD and in particular the degree of behavioral impairment [25, 26]. Altogether it is hypothesized that an imbalanced GI mucosal dysbiosis and immune dysfunction during a critical neurodevelopmental window pave the way for “leaky gut” and establish a chronic systemic and neuronal inflammatory setting [22, 23]. These data could explain the particular vulnerability of ASD subjects to intercurrent microbial infections with GI disturbances [27] and is consistent with the following observations: i) Intestinal microbiota composition in ASD subjects differ, both in number and diversity, from those in healthy control subjects [17]; ii) Monocytes from ASD^GI^ patients in culture exhibited innate immune response abnormalities including decreased production of both pro- and anti-inflammatory cytokines and increased expression of chemokine transcripts [27].

Even if the proof of concept of direct involvement of GI microbiome in ASD remains to be firmly established [28], given the above discussed published data, implication of altered genetic control of innate immune response processes to fungal and other microbial species in the etiopathology and/or severity of ASD is plausible. These immune processes intimately control the host/microbial homeostasis, and are mainly driven by a set of pattern recognition receptors (PRR) such as Dectin-1, encoded by the *CLEC7A* gene on chromosome 12 (12p13.2). Present on the surface of dendritic cells, neutrophils and macrophages, Dectin-1 recognizes and interacts with the β-1,3-glucan molecules present in the cell wall of nearly all fungi [29]. Upon receptor-ligand interaction, intracellular signaling is triggered which in turn induces the expression of pro-inflammatory cytokines and chemokines and engages the T helper (Th) 1 and Th17 cells towards cytotoxic T-cell responses [30, 31]. Interestingly, in humans, two SNPs (*CLEC7A rs*2087178 A and *CLEC7A rs*16910631G), either as allele or haplotype, have been associated with medically-refractory ulcerative colitis (MRUC) [32]. This observation incited us to postulate that such genetic changes in *Dectin-1* could also influence the susceptibility to ASD-associated GI disturbances. To explore this possibility, herein we studied, in a case-control design, the influence of *CLEC7A* gene polymorphisms (*rs*2078178 and *rs*16910631) on ASD clinical categories with IQ as proxy for the GI status in ASD.

## Material and Methods

### Subjects and clinical assessments

This study was conducted in a sample of subjects with ASD enrolled in the PARIS (Paris Autism Research International Sibpair) cohort in specialized clinical neuropsychiatric centers established in France and in Sweden [33]. Diagnosis was based on comprehensive clinical evaluation by expert clinicians using DSM IV-TR criteria. Subjects were assessed with the Autism Diagnostic Interview-Revised (ADI-R) and most of them also with the Autism Diagnostic Observation Scale (ADOS). Cases were included only after a thorough clinical evaluation, including psychiatric and neuropsychological examination, standard karyotyping, and fragile-X testing, as well as brain imaging and EEG as required. The IQ evaluation was carried out with an age-appropriate Weschler scale (WPPSI, Wechsler Preschool and Primary Scale of Intelligence; WISC, Wechsler Intelligence Scale for Children; or WASI, Wechsler Abbreviated Scale of Intelligence). For the most severe and/or non-verbal patients, the Raven’s Standard Progressive Matrices were used to measure nonverbal IQ (NVIQ) and the Peabody Picture Vocabulary Test (PPVT-4th edition) to measure receptive vocabulary (RV). The healthy control (HC) group consists of clinically assessed unrelated healthy individuals, both enrolled under the previously published selective criteria [34, 35]. All controls and most of the individuals included were of European descent.

Written informed consent was obtained from all participants including caretakers/guardians on behalf of minors/children included in the study and the documents recorded and stored in each participant center (Paris and Gothenburg). The study was approved by a local Institutional Review Board (IRB) i.e. the “Comités de Protection des Personnes (CPP) Île-de-France, Hôpital Pitié-Salpêtrière 75013 Paris” for France and the “Sahlgrenska Academy Ethics committee, University of Gothenburg” for Sweden.

### *CLEC7A* genotyping

Genomic DNA was extracted from EDTA-treated peripheral blood samples or B-lymphoblastoid cell lines using the Nucleon BACC3 kit (GE HealthCare, Chalfont St Giles, UK). The genotyping of the two SNPs herein studied (intron 1 *rs*16910631G/A and intron 3 *rs*2078178 A/G) was performed using a TaqMan 5’-nuclease assay (Applied Biosystems, Foster City, CA, USA) with allele-specific fluorogenic oligonucleotide probes. The following pre-developed TaqMan assay genotyping kits were used: C_33748498_10 and C_1932439_10.

### Statistical analysis

Power calculation was used in order to detect a statistically significant difference between two proportions with specified levels of confidence (0.95) and effect size. Comparison of genotype and allele frequency between patients and controls were performed using the Chi-square testing. The *p* values (two tailed) were corrected (*pc*) using the Bonferroni method and findings were considered statistically significant for *pc* less than 0.05. Odds ratio (OR) and confidence interval 95% (CI95%) were calculated to assess the relative risk conferred by a specific allele, genotype or haplotype. Deviation, if any, from Hardy-Weinberg expectations was analyzed using the chi-square test. For haplotype reconstruction, PHASE software (version 2.1) was used. This Bayesian algorithm provides the most-likely pairs of haplotypes carried by each subject [36, 37]. The tests Kruskal-Wallis or Mann–Whitney were used for nonparametric analysis [distribution of *CLEC7A* genotypes and haplotypes according to intellectual quotient (IQ) values]. High-IQ patients were defined as those with IQ values greater than 80, while low-IQ as those with IQ values between 25 and 70. Linear regression analyses were performed to examine the relationship between IQ, *CLEC7A* genotype and diagnosis as the predictive variables. The model was QI transformed ~ Genotypes + diagnosis. IQ was square root transformed to fulfill the normality assumption required by the parametric procedure. By default, calculations assume that a two-tailed statistical test was used at a confidence level of 95%. All statistical analyses were performed using the free statistical package R.2.13 software.

## Results

A sample of 478 subjects with ASD (364 males and 102 females), with a mean age of 15.42 ± 9.71 years (mean ± SD) (3 to 60 years) was included and compared to 351controls (171 males and 160 females) with a mean age of 35.69 ± 15.47 years (range 4 to 64 years) (Table 1).

**Table 1:** Demographic and clinical data of ASD patients and healthy controls.

		ASD patients	Healthy Controls (HC)
**Mean Age years ± SD (range)**		15.42 ± 9.71 (3–60)	35.69 ± 15.47 (4–64)
**Sex**	Male	364 (78%)	171 (52%)
	Female	102 (22%)	160 (48%)
**DSM-IV TR Diagnosis**	Asperger	56 (14%)	
	CA	331 (83%)	
	PDD-NOS	13 (3%)	

ASD: autism spectrum disorders

SD: standard deviation

CA: Classical autism

PDD-NOS: pervasive developmental disorders not otherwise specified.

For the present study, power calculation was made using the data from Illiev *et al*, and a theoretical statistical power of 84.8% was obtained.

Allele, genotype and haplotype distributions of the two *CLEC7A* polymorphisms are summarized in Tables 2 to 5. For both genetic variations the observed genotype distribution satisfied the expected Hardy-Weinberg proportions.

**Table 2:** *CLEC7A* genotype and allele frequencies among patients and controls.

*CLEC7A* variant		ASD		HC					
		n	%	n	%	p	p_c_	OR	95% CI
*rs*2078178	**Genotype**								
	GG	253	53	212	61	0.03	0.06	0.74	0.55–0.98
	AA+AG	224	47	138	39				
*rs*2078178	***Allele***								
	A	275	29	161	23	0.008	0.01	1.36	1.08–1.71
	G	679	71	539	77				

ASD: autism spectrum disorders

HC: healthy controls

n: number

pc: corrected p-value

OR: odds ratio; 95%

CI: confidence interval 95%.

**Table 3:** *CLEC7A* alleles and genotypes distribution in patients with Asperger and other ASD types.

*CLEC7A* variant		Asperger		Other ASD					
		n	%	n	%	p	p_c_	OR	95% CI
*rs*2078178	**Genotype**								
	GG	39	71	214	51	0.006	0.01	2.37	1.25–4.68
	AA+AG	16	29	208	49				
*rs*2078178	***Allele***								
	A	20	18	255	30	0.01	0.02	1.95	1.16–3.41
	G	90	82	589	70				

Other ASD: Classical autism and PDD-NOS

n: number

pc: corrected p-value

OR: odds ratio; 95%

CI: confidence interval 95%.

**Table 4:** *CLEC7A* alleles and genotypes distribution in patients with classical autism, Asperger and PDD-NOS.

*CLEC7A* variant		Asperger		CA					
		n	%	n	%	p	p_c_	OR	95% CI
*rs*2078178	**Genotype**								
	GG	39	71	166	50	0.005	0.01	2.42	1.26–4.83
	AA+AG	16	29	165	50				
*rs*2078178	***Allele***								
	A	20	18	200	30	0.009	0.01	1.95	1.15–3.43
	G	90	82	462	70				
*CLEC7A* variant		**Asperger**		**PDD-NOS**					
		n	%	n	%	p	p_c_	OR	95% CI
*rs*2078178	***Genotype***								
	GG	39	71	48	53	0.03	0.06	2.18	1.02–4.79
	AA+AG	16	29	43	47				
*rs*2078178	***Allele***								
	A	20	18	55	30	0.02	0.04	1.95	1.06–3.67
	G	90	82	127	70				

CA: Classical autism

PDD-NOS: pervasive developmental disorders not otherwise specified

n: number

pc: corrected p-value

OR: odds ratio; 95%

CI: confidence interval 95%.

**Table 5:** Haplotypes distribution in patients with classical autism and Asperger and controls.

Chr12 *SNP1-SNP2*	*Genotype*	Asperger		Other ASD					
		n	%	n	%	p	p_c_	OR	95%CI
	G-G/G-G	36	80	199	54	0.001	0.002	3.38	1.54–8.19
	A-G/A-G + A-G/G-G	9	20	168	46				
	***Genotype***	**Asperger**		**CA**					
		n	%	n	%	p	p_c_	OR	95%CI
*rs*2078178-*rs*16910631	G-G/G-G	36	80	154	53	0.001	0.002	3.48	1.57–8.5
	A-G/A-G + A-G/G-G	9	20	134	47				
	***Genotype***	**Asperger**		**PDD-NOS**					
		n	%	n	%	p	p_c_	OR	95%CI
	G-G/G-G	36	80	45	57	0.01	0.02	3.02	1.21–8.06
	A-G/A-G + A-G/G-G	9	20	34	43				

CA: Classical autism

Other ASD: Classical autism and pervasive developmental disorders not otherwise specified (PDD-NOS)

HC: healthy controls

n: number

pc: corrected p-value

OR: odds ratio; 95%

CI: confidence interval 95%.

We found that the *CLEC7A rs*2078178 wild type G allele and GG genotype were more frequent in HC as compared to individuals with ASD, although the statistical significance was borderline for the GG genotype association after correction (G allele: 77% *vs*. 71%; p = 0.008, pc = 0.01, OR = 1.36, [CI95%] = 1.08–1.71; GG genotype 61% *vs*. 53%; p = 0.03, pc = 0.06; OR = 0.74, [95%CI] = 0.55–0.98 for controls and patients respectively) (Table 2). The distribution of alleles and genotypes of the *CLEC7A rs*16910631 polymorphism did not significantly differ between patients and HC.

We then analyzed the distribution of these two genetic variations according to clinical specifiers of ASD i.e. classical autism, Asperger and PDD-NOS. We found that the *CLEC7A rs*2078178 G allele and GG genotype were more frequent among Asperger patients as compared to other ASD types (classical autism and PDD-NOS) (G allele and GG genotype: 82 *vs*. 70%, p = 0.01, pc = 0.02, OR = 1.95, [CI95%] = 1.16–3.41 and 71 *vs*. 51%, p = 0.006, pc = 0.01 OR = 2.37, [CI95%] = 1.25–4.68 in Asperger and non-Asperger respectively) (Table 3).

A further distinction of non-Asperger groups allowed us to confirm the Asperger-related *CLEC7A rs*2078178 G allele and GG genotype signature (G allele and GG genotype: 82 *vs*. 70%, p = 0.009, pc = 0.01, OR = 1.95, [CI95%] = 1.15–3.43 and 71 *vs*. 50%, p = 0.005, pc = 0.01 OR = 2.42, [CI95%] = 1.26–4.83 in Asperger and classical autism respectively; 82 *vs*. 70% p = 0.02, pc = 0.04, OR = 1.95, [CI95%] = 1.06–3.67 and 71 *vs*. 53%, p = 0.03, pc = 0.06 OR = 2.18, [CI95%] = 1.02–4.79 in Asperger and PDD-NOS respectively (Table 4).

Phased polymorphic profiles allowed us to identify three common haplotype-based genotypes *viz* the *CLEC7A rs*2078178/*rs*16910631 A-G/A-G, A-G/G-G and G-G/G-G genotypes. Further analysis confirmed the previous findings in that the *CLEC7A* G-G/G-G genotype is more prevalent in the Asperger group (*CLEC7A* G-G/G-G *vs*. other, 80% *vs*. 54%, p = 0.001, pc = 0.002, OR = 3.38, [CI95%] = 1.54–8.19) (Table 5).

**Fig 1:**
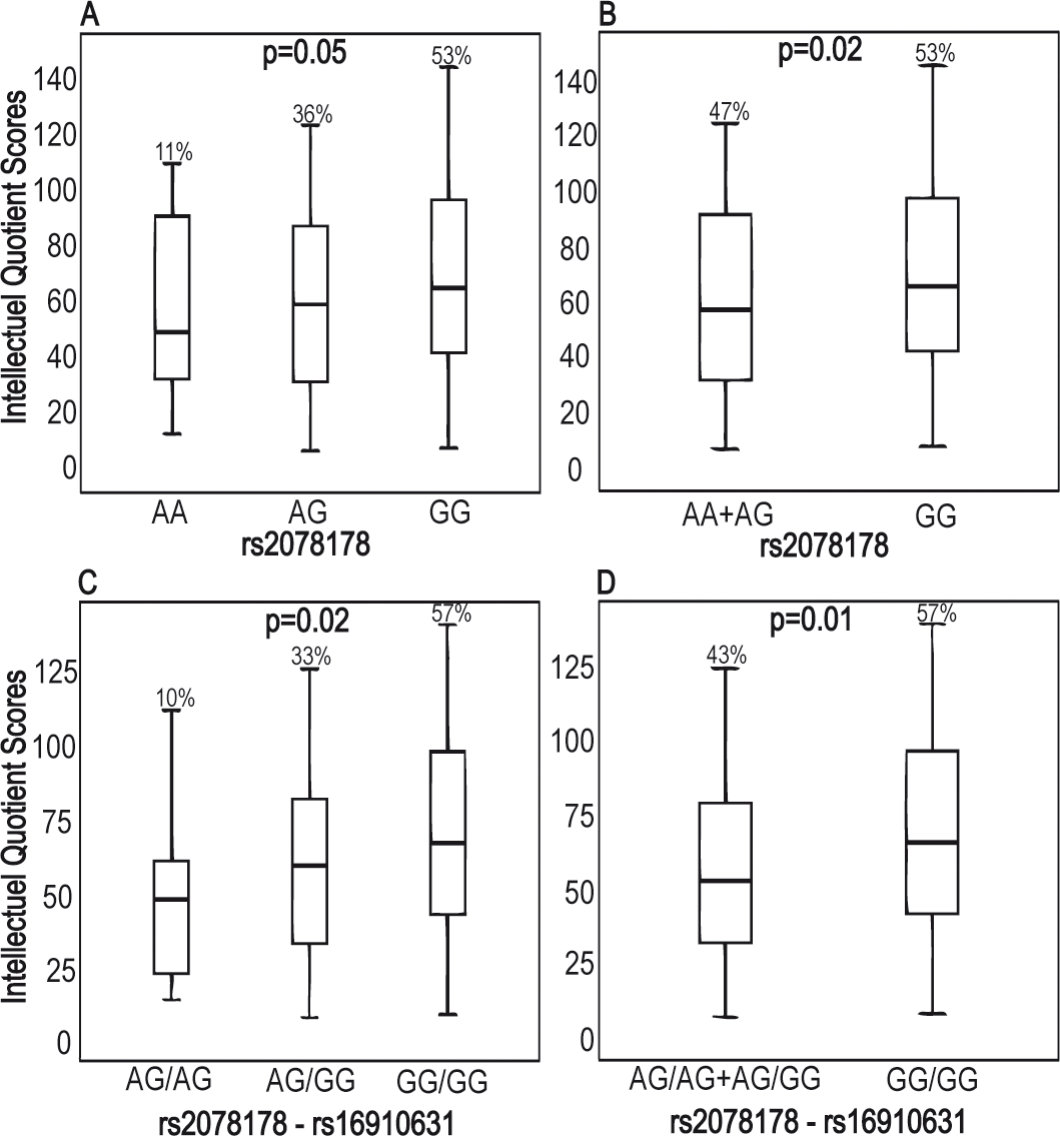
CLEC7A genotype and haplotype are associated with intellectual quotient (IQ) scores in ASD. A and B Kruskall-Wallis nonparametric testing show associations between *CLEC7A* genotypes (p = 0.05) / haplotypes (p = 0.02) and IQ scores. Patients bearing the *CLEC7A rs*2078178 GG or the *CLEC7A rs*2078178/*rs*16910631 G-G/G-G genotypes have higher IQ scores as compared to other patients. C and D Mann-Whitney nonparametric testing also showed significant associations for similar comparisons (respectively p = 0.02 and p = 0.01). The dark line inside the boxes represents the median value for each group. Boxes include the 25th and 75th quartiles; bars outside the boxes represent the maximal and minimal values.

As disease specifiers are expected to be associated with functional grade, at least in terms of degree of the accompanying intellectual disability, we analyzed the distribution of the *CLEC7A* genotype frequencies according to the IQ score distribution pattern. We found that mean IQ values exhibit increasing trend along the *CLEC7A* genotype groups: *rs*2078178 AA, AG and GG genotypes with respective IQ mean scores 55.7/ 59.3/ 67.4 (p = 0.05) with similar trend for *CLEC7A rs*2078178 GG *vs*. AA+AG genotypes (p = 0.02) (Fig 1). These data were further confirmed and strengthened by haplotype-based genotypes *CLEC7A* (genotype distribution frequencies according to IQ score distributions: *CLEC7A rs*2078178/*rs*16910631 A-G/A-G, A-G/G-G and G-G/G-G with p = 0.02; and *CLEC7A* G-G/G-G *vs*. others with p = 0.01) (Fig 1).

## Discussion

Given the now well documented immune/inflammatory substratum in ASD, genetically altered GI-tract dysbiosis is postulated to contribute to exacerbated inflammatory processes that could distinguish patient subsets with distinct clinical/behavioral phenotype. To explore this aspect, we opted to study the genetic polymorphisms of *CLEC7A* gene encoding Dectin-1 protein, a key molecule involved in fungal-mediated signaling and GI disorders, a common associated condition in ASD. We noted that

1. *CLEC7A* gene *per se* is not associated with susceptibility to ASD
2. *CLEC7A rs*2078178 G allele and GG genotype behave as genetic specifiers of Asperger among ASD
3. A two SNP-based (*rs*2078178-16910631) *CLEC7A* haplotype analysis confirmed the above finding.
4. Genetic association between this polymorphic locus with objectively assessed IQ values corroborates perfectly with the notion that *CLEC7A* is a genetic specifier in ASD.

These findings are in line with a recent study demonstrating the role of Dectine-1 in mediating ulcerative colitis severity [32]. Indeed, through functional studies in mice and genetic association studies in human, the authors demonstrated that: i) The susceptibility to severe colitis is genetically driven; (ii) Deficiency of Dectine-1 in mice and human cells promotes fungal infection; (iii) Induced colitis in *CLEC7A* knockout mice results in anti-*Saccharomyces cerevisiae* antibody production, the latter often observed in schizophrenia, another major psychiatric disorder; iv) In humans, *CLEC7A rs*2087178 A allele and *CLEC7A rs*2087178A-*rs*16910631G haplotype is associated with medically-refractory ulcerative colitis (MRUC). The demonstration that the susceptible A-G haplotype is associated with MRUC group, not with the treatment-responsive UC (TRUC) group, favors the notion that *CLEC7A* behaves as a disease modifier rather than a disease risk locus, a picture similar to what we captured in this study with respect to ASD. Dectin-1 also cooperates with various Toll-like receptors (TLRs) to enlarge the repertoire of antimicrobial defense [38, 39]. Indeed we recently described genetic associations between *TLR2* and *TLR 4* “low expressor” genotypes and bipolar disorder. This further strengthens the so far generated clues that genetically-driven deficient anti-infectious response during a vulnerable ontogenic/neurodevelopmental window may contribute to diverse psychiatric clinical categories and their clinical subsets [34, 40].

In ASD, behavioral symptoms and impaired cognitive skills are often accompanied by various comorbidities, with GI symptoms being the most common and worsening GI symptoms aggravate behavioral symptoms [24, 41, 42, 43].

These findings from chronic inflammatory settings in GI corroborate with our findings in ASD in that the *CLEC7A* AG haplotype, associated with inefficient response and altered immunity is less represented in high functioning Asperger ASD subgroup. This is understandable because the ASD^GI^ subgroup relatively runs a more severe course and often low functioning with significant behavioral disturbances. Then the question is why no genetic influence of CLEC7A was observed in genome wide association studies (GWAS) both in the context of inflammatory bowel disorder and ASD. As elegantly stated by Iliev et al [32], GWAS study design is such that it essentially uncovers the susceptibility genes, not those that influences the severity of the disease.

However, the important limitation of our retrospective study is absence of any information regarding the GI status in our patients but this study is an invitation to thoroughly explore the role of genetics of immune defense processes (TLRs, *CLEC7A*, *MHC)* in GI symptoms/dysfunction in ASD by studying large trans-geographic ASD cohorts well defined for potential environmental stressors, GI and IQ phenotypes.
