# Correction: Dectin-1 Polymorphism: A Genetic Disease Specifier in Autism Spectrum Disorders?

**DOI:** 10.1371/journal.pone.0214104

**Published:** 2019-03-14

**Authors:** Meriem Bennabi, Richard Delorme, José Oliveira, Catherine Fortier, Mohamed Lajnef, Wahid Boukouaci, Jean-Paul Feugeas, François Marzais, Alexandru Gaman, Dominique Charron, Bijan Ghaleh, Rajagopal Krishnamoorthy, Marion Leboyer, Ryad Tamouza

In the Subject and clinical assessments subsection of the Material and Methods, there is an error in the first sentence of the first paragraph. The study on Dectin polymorphisms in Autism Spectrum Disorders was not performed under the cadre of the Paris Autism Research International Sib-pair Study (PARIS study). Rather, the study was performed in the framework of a French project called, “Genetics of Autism (c07-33-2008-a00019-46) (PI Marion Leboyer),” which was approved by the local Institutional Review Board (IRB), the “Comités de Protection des Personnes (CPP) Île-de-France, XI, Saint Germain-en-Laye, France.”
